# Analysis of ventilator-associated pneumonia infection route by genome macrorestriction-pulsed-field gel electrophoresis and its prevention with combined nursing strategies

**DOI:** 10.3892/etm.2014.1994

**Published:** 2014-09-30

**Authors:** XIAODONG WANG, JUNPING WANG, JING LI, JING WANG

**Affiliations:** 1 Department of Pulmonary and Critical Care Medicine, The First Affiliated Hospital of Zhengzhou University, Zhengzhou, Henan 450052, P.R. China; 2 Department of Nursing, The First Affiliated Hospital of Zhengzhou University, Zhengzhou, Henan 450052, P.R. China

**Keywords:** ventilator-associated pneumonia, interventions, intensive care unit, genome macrorestriction-pulsed-field gel electrophoresis

## Abstract

The aim of the present study was to explore the infection route of ventilator-associated pneumonia (VAP) and assess the effectiveness of a combined nursing strategy to prevent VAP in intensive care units. Bacteria from the gastric juice and drainage from the hypolarynx and lower respiratory tracts of patients with VAP were analyzed using genome macrorestriction-pulsed-field gel electrophoresis (GM-PFGE). A total of 124 patients with tracheal intubation were placed in the intervention group and were treated with a combined nursing strategy, comprising mosapride (gastric motility stimulant) administration and semi-reclining positioning. A total of 112 intubated patients were placed in the control group and received routine nursing care. The incidence rate of VAP, days of ventilation and mortality rate of patients were compared between the two groups. The GM-PFGE fingerprinting results of three strains of *Pseudomonas aeruginosa* from the gastric juice, subglottic secretion drainage and drainage of the lower respiratory tract in patients with VAP were similar across groups. The number of days spent on a ventilator by patients in the intervention group (7.37±5.32 days) was lower compared with that by patients in the control group (12.34±4.98 days) (P<0.05). The incidence rate of VAP was reduced from 40.81 to 21.25% following intervention with the combined nursing strategy (P<0.05); furthermore, the mortality rate of intubated patients in the intervention group was 29.46%, a significant reduction compared with the 41.94% mortality rate observed in the control group (P<0.05). Gastroesophageal reflux (GER) was confirmed as one of the infection routes for VAP. The combined nursing strategy of gastric motility stimulant administration and the adoption of a semi-reclining position was effective in preventing VAP by reducing the occurrence of GER.

## Introduction

Ventilator-associated pneumonia (VAP) is one of the most common hospital-acquired pneumonias occurring in intubated patients and remains a leading cause of morbidity and mortality of patients in intensive care units (ICUs). Ventilator dependence and long hospital stays increase the costs of hospitalization. A number of risk factors exist for VAP, with bacterial colonization of the gastric content with subsequent gastroesophageal reflux (GER) and aspiration into the airways being an important risk factor (1). There is currently no particular method of preventing VAP; however, there are several promising combined nursing strategies that are effective in preventing VAP, including education programs, oral care and the continuous control of endotracheal cuff pressure and microaspiration of gastric contents. Dodek *et al* demonstrated that appropriate positioning to reduce reflux, improving oral hygiene, may reduce the incidence of VAP (2). In the present study, evidence for a retrograde route of VAP transmission from the stomach to the oropharynx to the lower respiratory tract was examined. Genome macrorestriction-pulsed-field gel electrophoresis (GM-PFGE) was performed to accomplish this and to subsequently assess the effects of a combined intervention strategy, comparing the mean risk of VAP between the control and intervention groups.

## Materials and methods

### Diagnosis of VAP

At present, the definition of VAP is controversial as it is difficult to distinguish the condition from other usual pulmonary infections. According to the definition by the American Thoracic Society and the Infectious Diseases Society of America, VAP is considered to be pneumonia in patients that have received mechanical ventilation for ≥48 h, characterized by the presence of a new or progressive infiltrate and signs of systemic infection (including temperature, blood cell count, changes in sputum characteristics and detection of the causative agent) (3). The condition can be divided into late-onset VAP (LOP) and early-onset VAP (EOP), depending on whether time spent on the ventilator was >5 or <5 days, respectively. In the present study, the clinical diagnosis criteria for VAP (positive quantitative endotracheal aspirate cultures and Clinical Pulmonary Infection Score >6) were based on the Hospital-Acquired Pneumonia Diagnosis and Treatment Guidelines by The Chinese Medical Association Branch of Respiratory Diseases (4).

A diagnosis of VAP (excluding certain associated lung diseases, including tuberculosis, lung cancer and atelectasis) was made if the condition of the patient met the following criteria: i) Lung infection following mechanical ventilation for 48 h; ii) presence of infiltrates or new inflammatory pulmonary lesions following mechanical ventilation; and iii) lung consolidation and/or moist lung rales, as well as one of the following: a) Blood cells >1.0×10^10^/l or <4×10^9^/l, with or without nuclear transfer; b) fever (body temperature >37.5°C) with a large number of purulent respiratory secretions; or c) new pathogenic bacteria isolated from bronchial secretions.

### Genotyping by GM-PFGE

Respiratory secretions from four patients with VAP [randomly selected from the ICU of the First Affiliated Hospital of Zhengzhou University (Zhengzhou, China)] were collected using protected specimen brushes once every other day for quantitative bacterial culture. The bacterial stains that antimicrobial susceptibility testing showed to be undifferentiated were cultured by centrifugation in lysogeny broth (Beijing Solarbio Science & Technology Co., Ltd, Beijing, China) at 37°C and 4.48 × g to a concentration of 10^9^/ml after 18 h. The cells were suspended in 100 μl buffer (0.1 mol/l EDTA, pH 8.0; 0.01 mol/l Tris-Cl, pH 7.6; and 1 mol/l NaCl), mixed with 100 μl low-melting point agarose (Sangon Biotech Co., Ltd., Shanghai, China) and molded into plugs at 4°C. Following congealing of the agar gel, the cells were lysed with lysis buffer at 37°C for 2 h and washed with Sodium Chloride-Tris-EDTA (STE) buffer (0.1 mol/l NaCl;10 mmol/l Tris-Cl, pH 8.0; and 1 mmol/l EDTA, PH 8.0), followed by enzymolysis with proteinase K (1 mg/ml; Sangon Biotech Co., Ltd.) for 16–20 h. The lysed cells were subsequently treated with 2 mmol/l phenylmethanesulfonyl fluoride (Beijing Cowin Bioscience Co., Ltd., Beijing, China) for 45 min; this treatment was repeated once more, prior to washing three times with STE buffer and mixing at room temperature with a buffer containing 50 units *Spe*I restriction enzyme (Beijing Cowin Bioscience Co., Ltd.). After 18 h at 37°C, electrophoresis was performed using GenePath (Bio-Rad, Hercules, CA, USA) at a field strength of 6 V/cm, at 14°C for 20 h. The pulse times were between 5 and 35 sec. The gel was then stained with ethidium bromide (0.5 μg/ml; HaoSen Co., Jiangsu, China) for 1 h and washed for 1 h with ddH_2_O. Finally, the gel was observed under a 302 nm ultraviolet light and images were captured. Fragment patterns were compared according to the criteria set out by Tenover *et al* (5).

### Study population

#### Inclusion and exclusion criteria

The inclusion criteria for the patients observed in the present study were as follows: i) >60 years old and ii) receiving mechanical ventilation. The exclusion criteria comprised: i) a critical condition that could cause mortality within 48 h; ii) mechanical ventilation for <48 h; and iii) pulmonary infection.

#### Intervention group

The 124 intubated patients (intervention group) were treated at the ICU of the First Affiliated Hospital of Zhengzhou University between January 2011 and May 2013. The intervention treatment included the administration of a gastric motility stimulant and the adoption of a semi-reclining position. Mosapride citrate tablets (5 mg/tablet; Lunan Company, Shandong, China) at a dosage of 5 mg/administration, three times a day, were selected as the gastric motility stimulant. The stimulant was used continuously until the study end-point or unless severe diarrhea (loose bowel movements three times in one day) occurred.

The semi-reclining position was used as a treatment if there were no tolerance issues or anti-semi-reclining indicators (including use of a vascular active drug, refractory shock following hypervolemic therapy inefficiency, or neurosurgery or abdominal surgery within the previous seven days). The beds of able patients were positioned at an angle of 30–45° to maintain these patients in a semi-reclining repose; for those patients that exhibited tolerance issues, a semi-reclining position was maintained during nasogastric feeding (including gastrointestinal injections), as well as for 2 h after feeding.

#### Control group

A total of 112 intubated patients received traditional nursing in the ICU of the First Affiliated Hospital of Zhengzhou University between January 2009 and December 2010. The patients were not administered any gastric motility stimulants during the treatment period.

All other procedures in the treatment of the two groups of patients were consistent and included the following: i) Monitoring of intra-cuff pressure every 4 h and maintenance at >20 mmHg, as well as the continuous aspiration of subglottic secretion, sputum smear, Gram staining and aerobic cultivation twice per week; ii) administration of sucralfate tablets; and iii) no use of H_2_ receptor antagonists and antacids unless bleeding of the stress ulcers occurred. In addition, Acute Physiology and Chronic Health Evaluation II (APACHE II) score of all patients were evaluated. APACHE II was an indicator of illness severity, which was determined using the worst value obtained during the initial 24 h following ICU admission, as well as on the day of VAP diagnosis (6,7).

Written informed consent was obtained from all patients prior to their involvement in the study. The study was approved by the Life Sciences Institutional Review Board of Zhengzhou University.

### Observation indices

A number of observations were recorded, including baseline indices (characteristics of the patients prior to treatment), length of hospital stay and number of days spent on the ventilator. In addition, GER was monitored in all the patients. The observation was concluded when one of the following occurred: Mortality, extubation or diagnosis of VAP with a one month follow-up to confirm whether the cause of the patient’s mortality was associated with VAP. The incidence rate of VAP referred to the number of VAP episodes per 1,000 ventilator-days. Mortality rates for the patients with and without VAP were calculated and compared between the two groups of patients.

### Statistical analysis

All statistical data were analyzed using SPSS 12.0 software (SPSS, Inc, Chicago, IL, USA). Rate data were calculated using a χ^2^ test. Significant differences between the two groups with one variant were determined using the Student’ t-test. A two-tailed value of P<0.05 was considered to indicate a statistically significant difference.

## Results

### GM-PFGE fingerprinting

**Figure 1:**
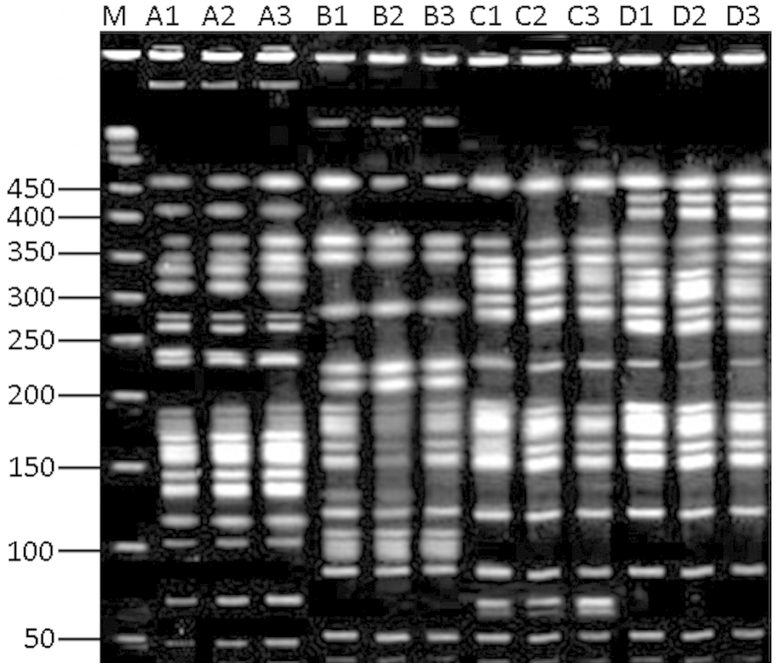
Genome macrorestriction-pulsed-field gel electrophoresis for 12 strains of *Pseudomonas aeruginosa* from four patients with VAP. M indicates the molecular size markers (λ ladder DNA). The letters A, B, C and D indicate the four patients with VAP. The numbers refer to the origin of the pathogens: 1, gastric juice; 2, subglottic secretion drainage and 3 drainage from the lower respiratory tract. VAP, ventilator-associated pneumonia.

The primary pathogen, *Pseudomonas aeruginosa*, was selected for genotyping using GM-PFGE. GM-PFGE fingerprinting indicated that the *P. aeruginosa* from the gastric juice, subglottic secretion drainage and drainage of the lower respiratory tract were similar in each patient with VAP (Fig. 1). However, while the strains were consistent across locations in a single patient, the strains observed across the four patients with VAP differed.

### Basic participant information

All patients in the intervention and control groups were selected strictly in accordance with the inclusion and exclusion criteria. The age, gender and basic data of the patients were normalized between the two groups. Furthermore, all patients were treated in the same hospital and the number of patients who were from medical or surgical departments was also normalized across the groups (Table I).

### Etiology of VAP

The most commonly detected bacteria were *P. aeruginosa* (29.42%), *Acinetobacter baumannii* (10.85%), *Staphylococcus aureus* (7.48%) and *Stenotrophomonas maltophilia* (7.32%). The number of patients with VAP infected with two or more bacteria was 55.81%. The primary pathogens identified in patients with EOP were Gram-positive bacteria (59.27%), including *S. aureus*, while Gram-negative bacteria (including *P. aeruginosa* and *A. baumannii*) were most frequently identified in patients with LOP (70.8%).

### Effect of intervention

Table II shows that the combined nursing strategy described in the present study was able to decrease the number of days spent on a ventilator, the incidence rate of VAP and the mortality rate of the intubated patients (all P<0.05); however, no significant difference in the ratio of EOP/LOP was found between the two groups. The mortality rate from VAP was not reduced by these nursing measures once it had occurred.

## Discussion

PFGE has been used as the gold standard for the genotyping of bacteria, which can be a powerful tool for the study of nosocomial infections. There is debate as to whether a retrograde route of transmission from the stomach to oropharynx to lower respiratory tract contributes to VAP. In the present study, GM-PFGE fingerprinting results demonstrated that this route does exist in patients with VAP. This result was consistent with that of a previous study (8) and may be the reason why the interventions in the present study were effective in the prevention of VAP. Grap *et al* (9) observed that an early, single application of chlorhexidine significantly reduced the occurrence of VAP in trauma patients. The results of the present study indicate that this may have been due to the chlorhexidine blocking the infection route from the stomach to respiratory tract, at least to a certain extent.

In the present study, the results of GM-PFGE genotyping indicated that the *P. aeruginosa* populations at different drainage locations in each patient were consistent. A previous animal study involving New Zealand white rabbits indicated that bacteria from the gastrointestinal tracts of the rabbits were not the main sources of EOP, but may have contributed to the development of LOP (10). Consequently, the prevention of VAP through inhibition of the gastroesophageal reflux (GER) may be effective.

Numerous studies have been performed to investigate the prevention of VAP, and several strategies have been proven to be effective preventative measures, including educational intervention (11,12), sufficient and professional oral care (13–15) and the use of specific equipment, such as heat and moisture exchange filters (16) or silver-coated endotracheal tubes (17). None of these measures, however, should be used as an isolated intervention.

The combined strategy used in the present study, which focused predominantly on body position and the inhibition of the occurrence of GER, revealed that a semi-reclining position and the use of mosapride (an inhibitor of GER) were able to decrease the incidence rate of VAP from 40.81 to 21.25% (P<0.05). The number of days spent on a ventilator was reduced by almost five days in the intervention group (P<0.05), and the mortality rate of intubated patients decreased from 41.94 to 29.46% (P<0.05). However, the interventions had no effect on the mortality rate following the occurrence of VAP, demonstrating that the effectiveness of these interventions lies entirely in preventing VAP onset. These results were consistent with those of a previously published study (18). An animal model of mechanical ventilation using healthy New Zealand white rabbits demonstrated that drugs promoting gastrointestinal motility (mosapride citrate) were useful in reducing the incidence rate of VAP caused by bacteria from the digestive tract (10).

As the development of VAP is a complicated process, its overall prevention requires numerous stages, courses and methods. Further studies are required to investigate the infection route from the stomach to the respiratory tract in patients with VAP, and more effective and personalized strategies for reducing episodes of VAP should be utilized.

## Figures and Tables

**Table I:** Basic participant information.

	Group		
		
Characteristic	Control	Intervention	P-value
n	112	124	-
Age in years, mean ± SD	70.63±6.31	71.06±5.74	0.851
Gender, n male/n female	67/57	59/53	0.764
APACHE II score, mean ± SD	22.84±5.75	24.84±4.96	0.672

APACHE II, Acute Physiology and Chronic Health Evaluation II; SD, standard deviation.

**Table II:** Comparison of several indexes between the control and intervention groups.

	Group		
		
Indexes	Control	Intervention	P-value
VAP, n (EOP/LOP)	84 (26/58)	43 (11/23)	0.882
Ventilator-days, mean ± SD	12.34±4.98	7.37±5.32	<0.050
Incidence of VAP^a^, ‰	40.81	21.25	<0.050
Mortality rate, %			
Intubated patients	41.94	29.46	<0.050
Patients with VAP	35.71	47.06	0.252

a The incidence of VAP means the number of VAP episodes per 1,000 ventilator-days.

VAP, ventilator-associated pneumonia; EOP, early-onset VAP; LOP, late-onset VAP; SD, standard deviation.
